# Inhibitory activities of essential oils from *Syzygium aromaticum* inhibition of *Echinochloa crus-galli*

**DOI:** 10.1371/journal.pone.0304863

**Published:** 2024-06-21

**Authors:** Xianzhi Ni, Haodong Bai, Jincai Han, Yong Zhou, Zhendong Bai, Siquan Luo, Jingjing Xu, Chenzhong Jin, Zuren Li

**Affiliations:** 1 Hunan Provincial Key Laboratory for Biology and Control of Weeds, Collaborative Innovation Center for Field Weeds Control, Science and Technology, Hunan University of Humanities, Loudi, China; 2 Key Laboratory of Pesticide Assessment, Ministry of Agriculture and Rural Affairs, P.R. China, Hunan Academy of Agricultural Sciences, Changsha, China; University of Brescia: Universita degli Studi di Brescia, ITALY

## Abstract

*Echinochloa crus-galli* is a serious weed species in rice paddies. To obtain a new potential bioherbicide, we evaluated the inhibitory activities of 13 essential oils and their active substances against *E*. *crus-galli*. Essential oil from *Syzygium aromaticum* (L.) Merr. & L. M. Perry (SAEO) exhibited the highest herbicidal activity (EC_50_ = 3.87 mg mL^-1^) among the 13 essential oils evaluated. The SAEO was isolated at six different temperatures by vacuum fractional distillation, including 164°C, 165°C (SAEO—165), 169°C, 170°C 175°C and 180°C. The SAEO—165 had the highest inhibitory rate against *E*. *crus-galli*. Gas chromatography-mass spectrometry and high phase liquid chromatography identified eugenol (EC_50_ = 4.07 mg mL^-1^), α-caryophyllene (EC_50_ = 17.34 mg mL^-1^) and β-caryophyllene (EC_50_ = 96.66 mg mL^-1^) as the three compounds in SAEO. Results from a safety bioassay showed that the tolerance of rice seedling (~ 20% inhibition) was higher than that of *E*. *crus-galli* (~ 70% inhibition) under SAEO stress. SAEO induced excessive generation of reactive oxygen species leading to oxidative stress and ultimately tissue damage in *E*. *crus-galli*. Our results indicate that SAEO has a potential for development into a new selective bio-herbicide. They also provide an example of a sustainable management strategy for *E*. *crus-galli* in rice paddies.

## Introduction

Weeds pose a growing menace to global food production [1]. Barnyard grass (*Echinochloa crus-galli*) is currently among the most economically damaging weeds in global agriculture [2, 3]. *E*. *crus-galli* releases a number of allelochemicals into paddy soils to suppress rice growth, but at a low rate [4]. Recently, a number of synthetic chemicals have been authenticated and applied to control the negative impacts of weeds on crops [5]. However, the extensive and repeated use of chemical herbicides, has greatly damaged the environment, but has also resulted in resistant barnyard grass accessions to herbicides, with different mechanisms of action [6, 7]. Consequently, warranting a novel and environmental-friendly weed management method.

Essential oils serve as sources of bioactive natural products, and they have high diversity, multitarget activity, biodegradable and show high sensitivity to organisms [8–10]. Therefore, these can provide a useful resources for weed management. At present, most of the studies conducted on plant extracts, have focused on their use as bacteriostatics, antioxidants and insecticides [11–13]. However, the phytotoxicity of essential oils can be used for weed control in agricultural production. Recently, different researchers have demonstrated the weed-suppression potential of various EOs [6, 14–16]. Plant EOs can be moderately considered as green pesticides for the continuble management of insect pests and diseases [17]. Thus, it is pertinent to investigate EOs from widely used plant species for their herbicidal activity and potential agricultural.

To screen a high variety of essential oils with bioactivities, we chose thirteen crude essential oils from plants species which grow abundantly in China. These essential oils were applied to treat *E*. *crus-galli* to assay their herbicidal effects. The high-effective oils were isolated by vacuum fractional distillation and their activities evaluated in bioassay experiments. The specific chemical constituents of these oils were identified by gas chromatography-mass spectrometry (GC-MS) and high performance liquid chromatography (HPLC) and their herbicidal activities were evaluated in bioassay experiments. We also evaluated the effect of one promising essential oil, SAEO on catalase (CAT), peroxidase (POD), superoxide dismutase (SOD) and malondialdehyde (MDA) in *E*. *crus-galli*. The study serves as a baseline for the development of new selective biological herbicides for the control of *E*. *crus-galli* in rice paddies.

## Materials and methods

### Material and culture conditions

The seeds of *E*. *crus-galli* and rice used in the experiment were collected from a rice field at Chunhua Base in Changsha city in Hunan Province, China (113°14’ 57″ E 28°16’ 49″N). Plants were grown in the greenhouse (12 h photoperiod (light / dark cycle), 110–140 μmol m^-2^ s^-1^ and 27 / 22°C (day / night temperature regime)). Spraying was done with a 3WP-2000 walking spray tower (sprayer delivered 225 L ha^-1^, walking speed 0.12–1.2 m/s, spraying pressure 15 pa) purchased from Nanjing Institute of Agricultural Mechanization, Ministry of Agriculture and Rural Affairs).

### Test plant essential oils

Thirteen plant essential oils were obtained by steam distillation. They included (*Mentha × piperita* Linnaeus essential oil (MPEO) # 1, *Syzygium aromaticum* (L.) Merr. & L. M. Perry essential oil (SAEO) # 2, *Eugenia caryophyllata* Thunb essential oil (ECEO) # 3, *Cinnamomum cassia* Thunb essential oil (CCEO) # 4, *Zingiber* boehm essential oil (ZEO) # 5, *Cymbopogon citratus* Presl essential oil (CIEO) # 6, *Cinnamomum camphora* (L.) Presl essential oil (CAEO) # 7, *Capsicum annuum linn*. var. grossum (L.) Sendt essential oil (CNEO) # 8, *Camellia sinensis* (L.) O. Ktze essential oil (CSEO) # 9, *Artemisia caruifolia* var. schochii essential oil (ACEO) # 10, *Citrus sinensis* Osb. var. brasliliensis Tanaka essential oil (CEEO) # 11, *Citrus limon* (Linnaeus) Osbeck essential oil # (CLEO) 12, and *Citrus reticulata* Blanco essential oil (CREO) # 13).

### Seed germination bioassay

Fifteen healthy rice and *E*. *crus-galli* seeds were germinated in Petri dishes (diameter = 9 cm) lined with a single layer of Whatman # 2 filter paper and grown in the greenhouse. Different concentrations of the essential oil, SAEO (pH = 5.0, essentially nonvolatile), were prepared (1, 2, 2.5, 4, 5 mg essential oil into 0.5 mL of Dimethylformamide (DMF), and into 10 mL of 6 / 1000 Tween 80 aqueous solution). Then 5 mL of each was added separately to the petri dishes, each which had fifteen rice seeds planted in them. Treatment solution without essential oil was used as control. Twenty *E*. *crus-galli* and rice seeds were also planted in Petri dishes lined with filter paper. All the Petri dishes were kept in a growth chamber set at 12 h photoperiod (light / dark cycle), 110–140 μmol m^-2^ s^-1^ and 27 / 22°C (day / night temperature regime). The number of seeds that germinated were recorded after seven days and the germination ratio of each group was calculated based on the data. The experiment was repeated three times. Parameters were calculated as follows: Germination percentage = (number ofgerminated seeds / total number of seeds) × 100 [18].

### Seedling growth bioassay

Fifteen healthy rice and *E*. *crus-galli* seedlings were grown in pots (volume = 9 × 9 cm) with 100 g of garden soil (content of organic matter ≤ 3%, pH = 5.42) of pearlite / manure (200 g L^-1^) mixture. Different concentrations of SAEO (*E*. *crus-galli*: 0.01, 0.05, 0.1, 0.2, 0.3 g, rice: 0.1, 0.3, 0.5, 0.8, 1 g essential oil into 0.5 mL of DMF, and into 10 mL of 6 / 1000 Tween 80 aqueous solution, pH = 5.0, essentially nonvolatile) was administered at the rate of 3.3 mL pot^−1^on the barnyard grass (2—leaf stage) and rice seedlings (2—leaf stage) and kept in the greenhouse. The plants were used in experiments when they were at the 2—leaf stage. Plant samples were harvested after seven days and their fresh weights recorded. Inhibition ratios of the essential oil were calculated based on the fresh weight data. The experiment was repeated three times. The seedlings inhibition ratio was calculated using the formula: [(Control-test sample) / Control × 100%] [19].

### Collection and isolation of essential oil by vacuum fractional distillation

The active substance in SAEO was obtained by isolation using vacuum fractional distillation [20]. The fractions of oil were obtained through a fractional distillation system with a pressure of—95 kPa from Minjie (Hunan, China). The distillation temperature range was 164 to 180°C(fraction Ⅰ temperature: 165°C, 170°C, 180°C; fraction Ⅱ temperature: 164°C, 169°C and 175°C). The collection period between each fraction was approximately 20 min.

Bioassays were then conducted with different concentrations (100, 300, 500 mg essential oil into 0.5 mL of DMF, and into 10 mL of 6 / 1000 Tween 80 aqueous solution) of the collected condensed SAEO (at 165°C, 170°C and 180°C). They were sprayed evenly on barnyard grass seedlings (2—leaf stage). Grass samples were harvested after seven days and their fresh weights recorded. Inhibition ratios of the SAEO were calculated based on the fresh weight data. The experiment was repeated three times. The experimental group with the highest inhibition ratio was selected for a further secondary distillation at 164°C, 169°C and 175°C. Bioassay experiments were then conducted as described before and the distillate collected. The experimental group which exhibited the highest inhibition ratio, was selected for gas chromatography-mass spectrometry (GC-MS) analysis.

### Gas chromatography-mass spectrometry (GC-MS) analysis

The chemical constituents of SAEO were identified using GC-MS. The data were acquired on a Shimazu GC-MS-QP2010 gas chromatograph (Shimazu, Japan). The chromatographic column conditions were as follows: the chromatographic column was RTX-5MS (30 m × 0.25 mmID × 0.25 μm df) and the carrier gas was highly pure helium, which had a flow rate of 0.8 mL min^-1^, a diversion ratio of 3.0 and vaporization temperature of 300°C. The temperature program was a starting temperature of 100°C, maintained at 0.0 min, a heating rate of 20.0°C min^-1^ to 180°C, maintained at 1.0 min, a heating rate of 5.0°C min^-1^ to 200°C, maintained at 1.0 min, and a heating rate of 20.0°C min^-1^ to 280°C, maintained at 4.0 min. The injection rate volume was 1 μL and the delay time of solvent was 3.15 min. Ion source: EI source, electron energy: 70 eV, ion source temperature: 240°C, interface temperature: 280°C, and scanning mass range: m / z 33–600. The nist05, nist05s and wiley7 libraries were used for identification of compounds. The relative amount of extract was determined using the peak area of the total ion chromatograms (TIC).

### Bioassay with major compounds

Toxicity tests were carried out using three major compounds identified in SAEO from the GC-MS (eugenol, β-caryophyllene and α-caryophyllene). Eugenol was purchased from Macklin Biochemical Co., Ltd (Shanghai, China). β-caryophyllene and α-caryophyllene were purchased from J&K Scientific Ltd. (Beijing, China). Different concentrations of the three compounds were prepared (10, 50, 100, 300, 500 mg added to the mixture of 0.5 mL of DMF, and into 10 ml of 6 / 1000 Tween 80 aqueous solution) and each sprayed on *E*. *crusgalli* seedlings at the 2—leaf stage. Grass samples were harvested after seven days and their fresh weight recorded. Inhibition ratios for each concentration were calculated based on the fresh weight data. The experiment was repeated three times.

### High performance liquid chromatography (HPLC)

The eugenol content in SAEO collected at 165°C (SAEO—165) was determined using HPLC, which was performed on a Shimazu LC-10AD pump and an SPD-10A UV detector, with a C_18_ column (4.6 mm × 200 mm, packing: Hypersil, particle size: 5 μm) from Dalian Elite Analytical Instruments. The acetonitrile and methanol used for the HPLC were chromatographic pure and the water was also aseptic. The chromatographic condition was (mobile phase: methanol and water (65: 35), flow rate: 1 mL min^-1^, detection wavelength: 280 nm, column temperature: 25, injection volume: 20 μL). The eugenol standard used was purchased from Shanghai Macklin Biochemical Co., Ltd (99% purity).

Preparation of standard: 20 mg of eugenol was weighed and added to the mobile phase, which was shaken and diluted to a concentration of 100 mg L^-1^. Preparation of test solution: SAEO—165 was dissolved in sterile water and diluted to a ratio of 1: 100, 000. A volume of 20 μl each of the reference and test solutions was injected into the liquid chromatograph and ran according to the above chromatographic conditions. The chromatogram and peak area were recorded and the content was calculated based on the peak area.

### Defense enzyme activities

Standard SAEO (0.5 g) was mixed with 0.5 mL of DMF and 10 ml of 6 / 1000 Tween 80 aqueous solution and sprayed on *E*. *crusgalli* and rice seedlings at the 2—leaf stage. Grass sample were collected at 1, 4, 8, 24, 48 and 72 h after spraying SAEO, and stored at—70°C until the measurement of defense enzymes activities, malondialdehyde (MDA) and plant total proteins. The activities of the antioxidant enzymes, superoxide dismutase (SOD), peroxidase (POD), catalase (CAT) and MDA were determined with kits following the manufacturer’s instructions. Total protein (TP) was determined using the Coomassie bright blue method. SOD, POD, CAT TP kits were purchased from Nanjing Jiancheng Bioengineering Institute. MDA kits were purchased from Beijing Solarbio Life Sciences.

### Statistical analyses

The data were analysed by one-way analysis of variance (ANOVA), and treatment values were compared at P ≤ 0.05. Data variance was determined using the data processing system (DPS) software and error analysis was carried out in MS Excel 2019. Graphs were produced using Origin 2017. The statistical analyses were performed using SPSS software (SPSS Inc., Chicago, Illinois, USA).

## Results

### SAEO inhibits the growth of *E*. *crus-galli*

**Fig 1:**
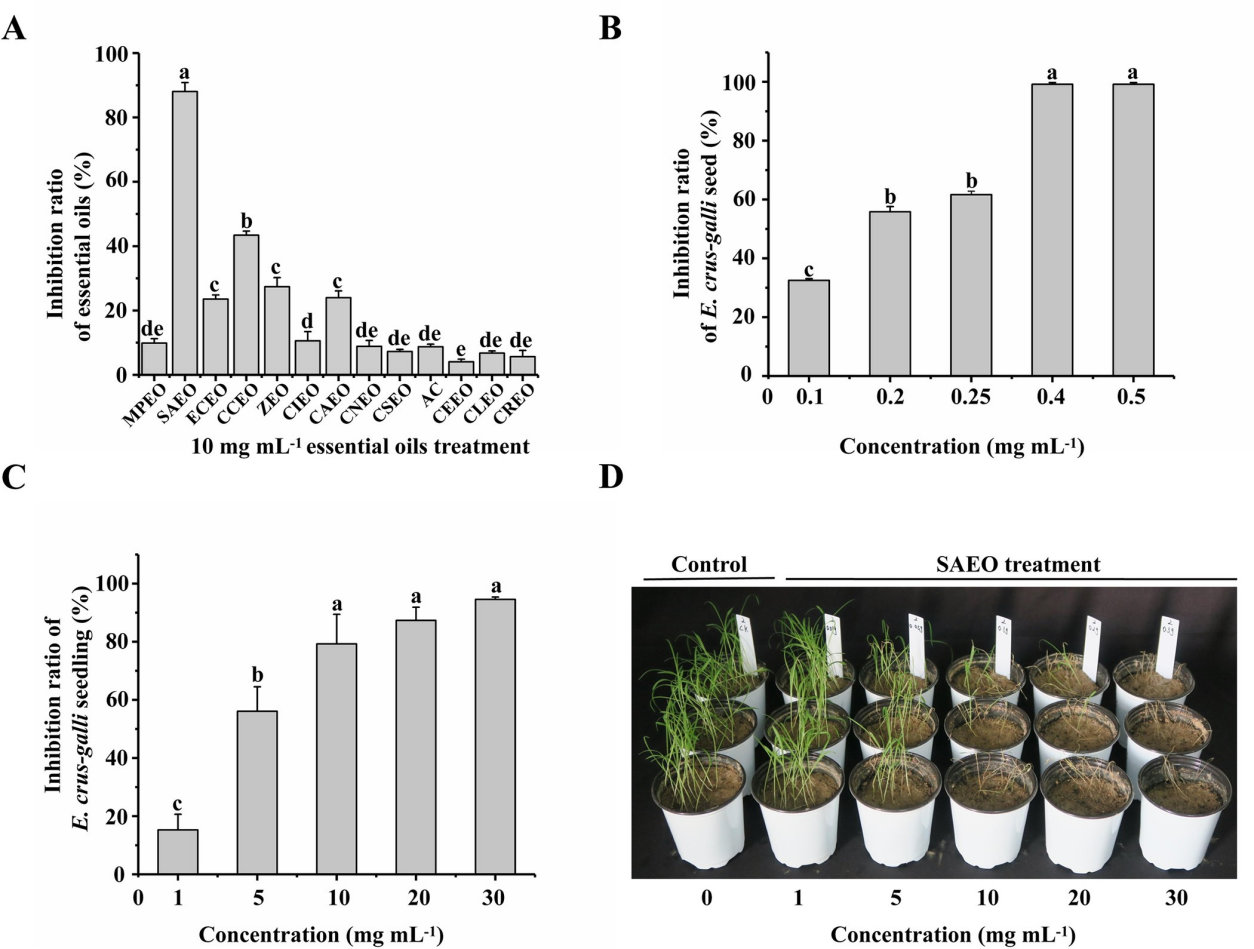
Bioassay results of essential oils on barnyardgrass. (**A**) Inhibition ratio of 13 essential oils (10 mg mL^-1^) on seedling growth of *E*. *crus-galli* 7 days after treatment. (*M*. *piperita* essential oil (MPEO), *S*. *aromaticum* essential oil (SAEO), *E*. *caryophyllata* essential oil (ECEO), *C*. *cassia* essential oil (CCEO), *Zingiber* essential oil (ZEO), *C*. *citratus* essential oil (CIEO), *C*. *camphora* essential oil (CAEO), *C*. *annuumlinn* essential oil (CNEO), *C*. *sinensis* essential oil (CSEO), *A*. *caruifolia* essential oil (ACEO), *C*. *sinensis* essential oil (CEEO), *C*. *limon* essential oil # (CLEO), and *C*. *reticulata* essential oil (CREO). The error bar is the standard error of the mean. The letter above the error bar indicates significant difference between means (ANOVA). The same letter indicates no significant differences between treatments. (**B**) Inhibitory effect of different SAEO concentrations (0.1, 0.2, 0.25, 0.4, 0.5 mg mL^-1^) on *E*. *crus-galli* seed. (**C**) Inhibitory effect of different SAEO concentrations (1, 5, 10, 20, 30 mg mL^-1^) on *E*. *crus-galli* seedling. (**D**) Inhibitory effect of different SAEO concentrations (0, 1, 5, 10, 20, 30 mg mL^-1^) on *E*. *crus-galli* seedlings growth.

The inhibitory effects of 13 essential oils on barnyard grass was studied in bioassay experiments. SAEO and CCEO had inhibitory effects on *E*. *crus-galli*, seven days after spraying seedlings with the 13 essential oils at 10 mg mL^-1^ (Fig 1A). The other 11 essential oils had no significant inhibitory effect (S1 Fig). Of the two, SAEO had more pronounced inhibitory effect. It showed an inhibition ratio of 86.46% on *E*. *crus-galli* seedlings at a concentration of 10 mg mL^-1^ (Fig 1A).

The inhibition ratio on seed germination was 33.33% when the concentration of SAEO was 0.1 mg mL^-1^, and 99.17% when the concentration was 0.4 mg mL^-1^ (Fig 1B). These two concentrations (0.4, 0.5 mg mL^-1^) caused near-complete inhibition (>99%) of seed germination. The results showed that the SAEO strongly inhibited seed germination. At the seedling stage (2—leaf stage), the inhibition ratio was 15.27% when the concentration of SAEO was 1 mg mL^-1^, and 94.56% when the concentration was 30 mg mL^-1^ (Fig 1C). The results showed that the SAEO inhibited seedling growth in a concentration-dependent way. The EC_50_ of SAEO at the seed germination stage of *E*. *crus-galli* was 0.1549 mg mL^-1^, and at the seedling stage was 3.87 mg mL^-1^ (S1 Table). The inhibitory effect significantly increased with increase in SAEO concentration (Fig 1D). The inhibitory effects of the 2 fraction distillated SAEO on *E*. *crus-galli* are shown in S2 Fig.

### SAEO components analysis by GC-MS and its bioassay

**Fig 2:**
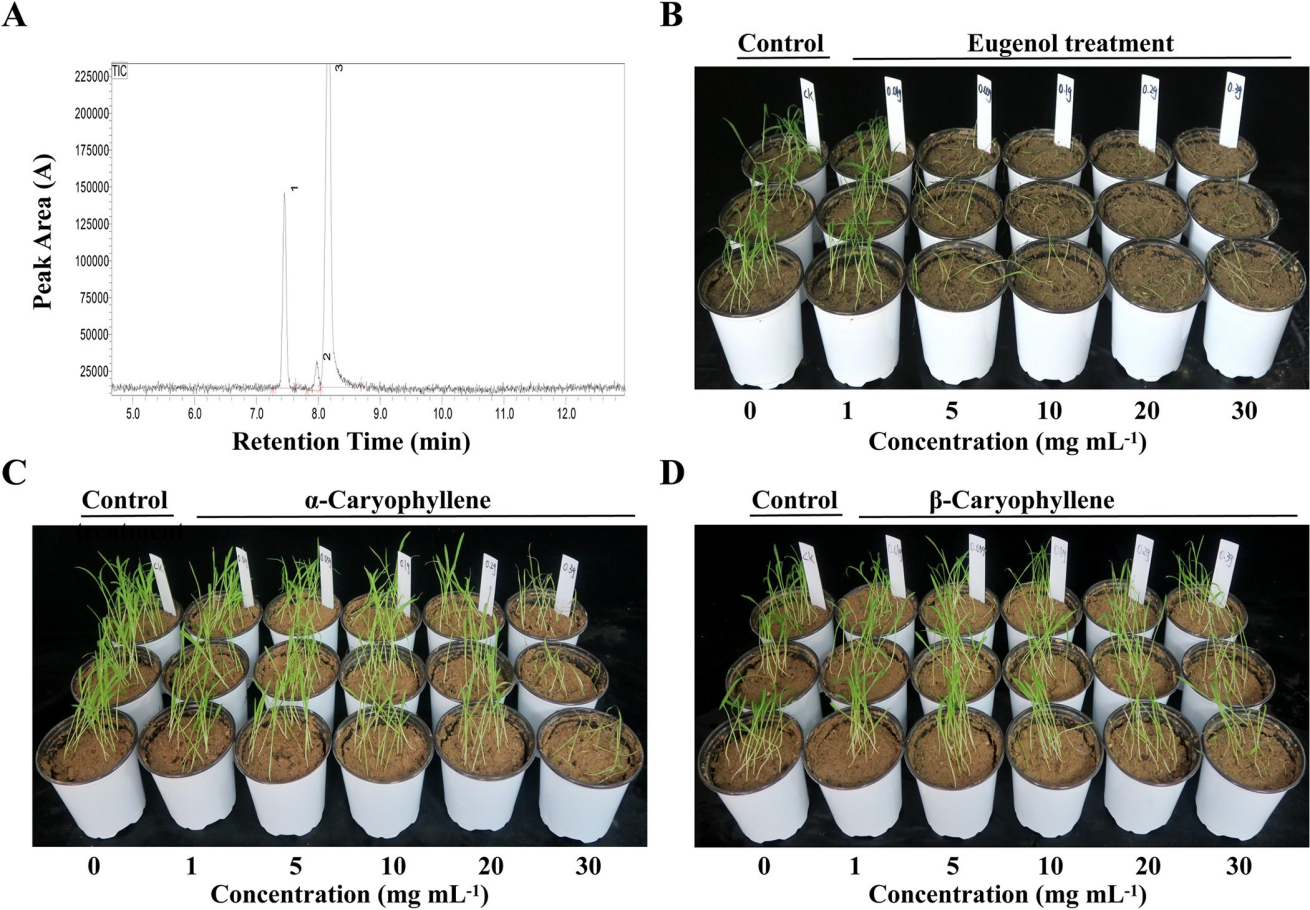
Main compounds identified in SAEO-165 by GC-MS and their inhibitory effects. (**A**) Diagram of GC-MS. (**B**) Inhibitory effect of different concentrations of eugenol (0, 1, 5, 10, 20, 30 mg mL^-1^) on *E*. *crus-galli*. (**C**) Inhibitory effect of different concentrations of α-caryophyllene (0, 1, 5, 10, 20, 30 mg mL^-1^) on *E*. *crus-galli*. (**D**) Inhibitory effect of different concentrations of β-caryophyllene (0, 1, 5, 10, 20, 30 mg mL^-1^) on *E*. *crus-galli*.

Three major chemical constituents were identified by GC—MS (Fig 2A) in SAEO—165 and these were eugenol (73.35%), β-caryophyllene (23%), and α-caryophyllene (3.65%) (Table 1). The EC_50_ of eugenol, α-caryophyllene and β-caryophyllene recorded at the seedling stage of *E*. *crus-galli* were 4.07 mg mL^-1^, 17.34 mg mL^-1^ and 96.66 mg mL^-1^, respectively (S2 Table). Eugenol, α-caryophyllene and β-caryophyllene showed significant inhibitory effect and their inhibition ratios recorded at a concentration of 5 mg mL^-1^ were 74.11%, 28.06% and 12.3%, respectively (Fig 2C and 2D). The inhibition ratios of the three compounds at 30 mg mL^-1^ were 88.69%, 69.65% and 40.95% respectively. Eugenol showed better herbicidal efficacy than β-caryophyllene and α-caryophyllene.

**Table 1:** The GC-MS analysis of SAEO-165.

Compounds	Retention Time	Integration Start Time	End Time of Integration	Peak Area (A)	Peak Area Percentage (A%)	RetIndex
β-caryophyllene	7.45	7.263	7.63	527982	23	1494
α-caryophyllene	7.973	7.817	8.033	83767	3.65	-
eugenol	8.168	8.033	8.693	1683438	73.35	-

### Vacuum fractional distillation and content identification

To identify the active fraction which can serve as a bioherbicide, we obtained purified SAEO by distillation (fraction Ⅰ temperature: 165°C, 170°C, 180°C; fraction Ⅱ temperature: 164°C, 169°C and 175°C, presstion: -95 kPa). The order of inhibitory effect for fraction I temperatures was 165°C > 170°C > 180°C and that for fraction Ⅱ was 164°C > 175°C > 169°C (S2 Fig).

**Fig 3:**
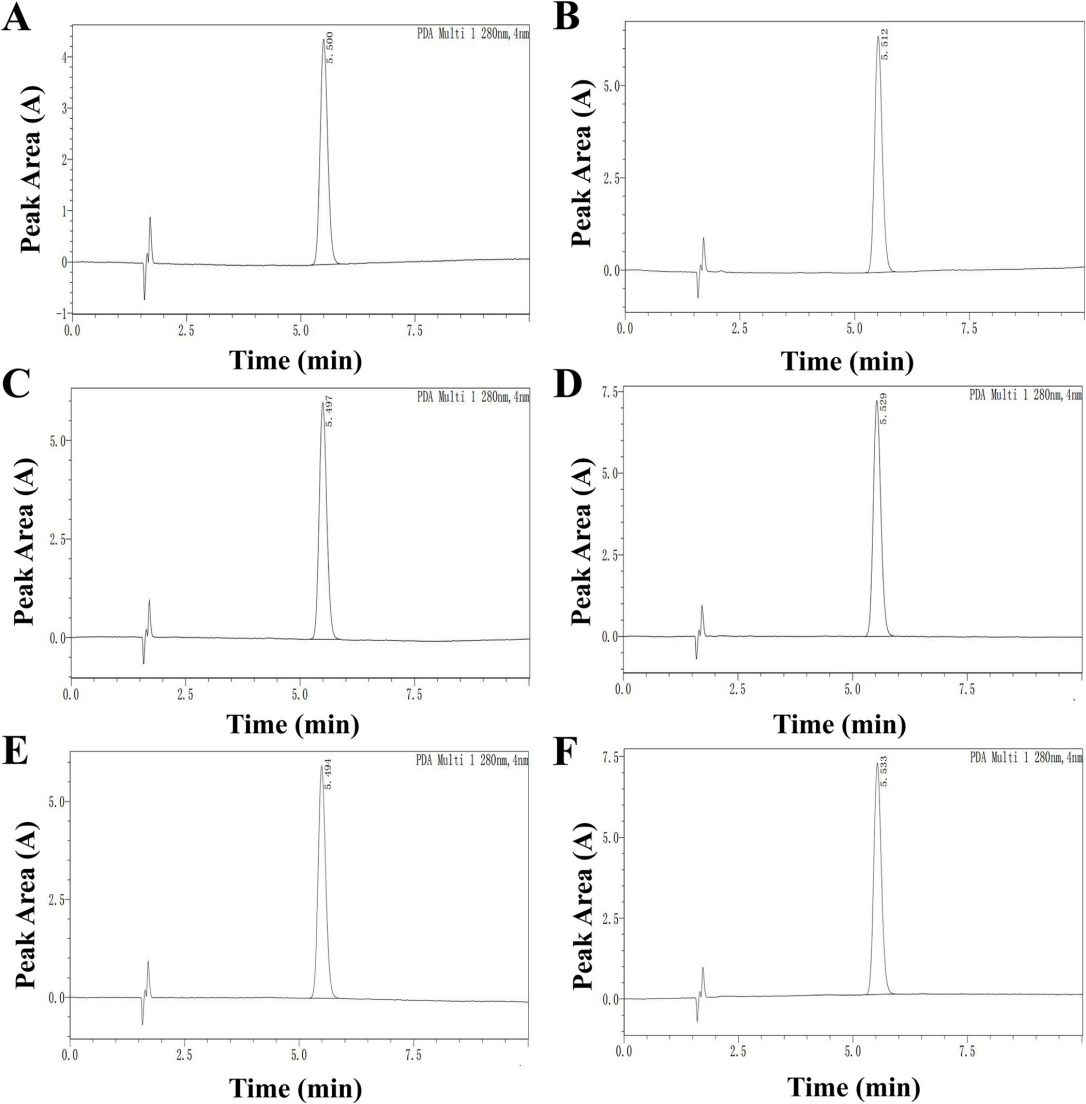
The eugenol chromatlog in SAEO conditions from HPLC analysis. (**A**) 164°C. (**B**) 165°C. (**C**) 169°C. (**D**) 170°C. (**E**) 175°C. (**F**) 180°C.

The HPLC results indicated that the peak area of eugenol purified from SAEO—164 was 50488 and its content was 50.488%, SAEO—165 was 73770 and its content was 74.77%, SAEO—169 was 68906 and its content was 68.906%, SAEO—170 was 83364 and its content was 83.364%, SAEO—175 was 67807) and its content was 67.807%, SAEO—180 was 82306 and its content was 82.306% (Fig 3, S3 Table).

### SAEO is relatively safe for rice seedlings

**Fig 4:**
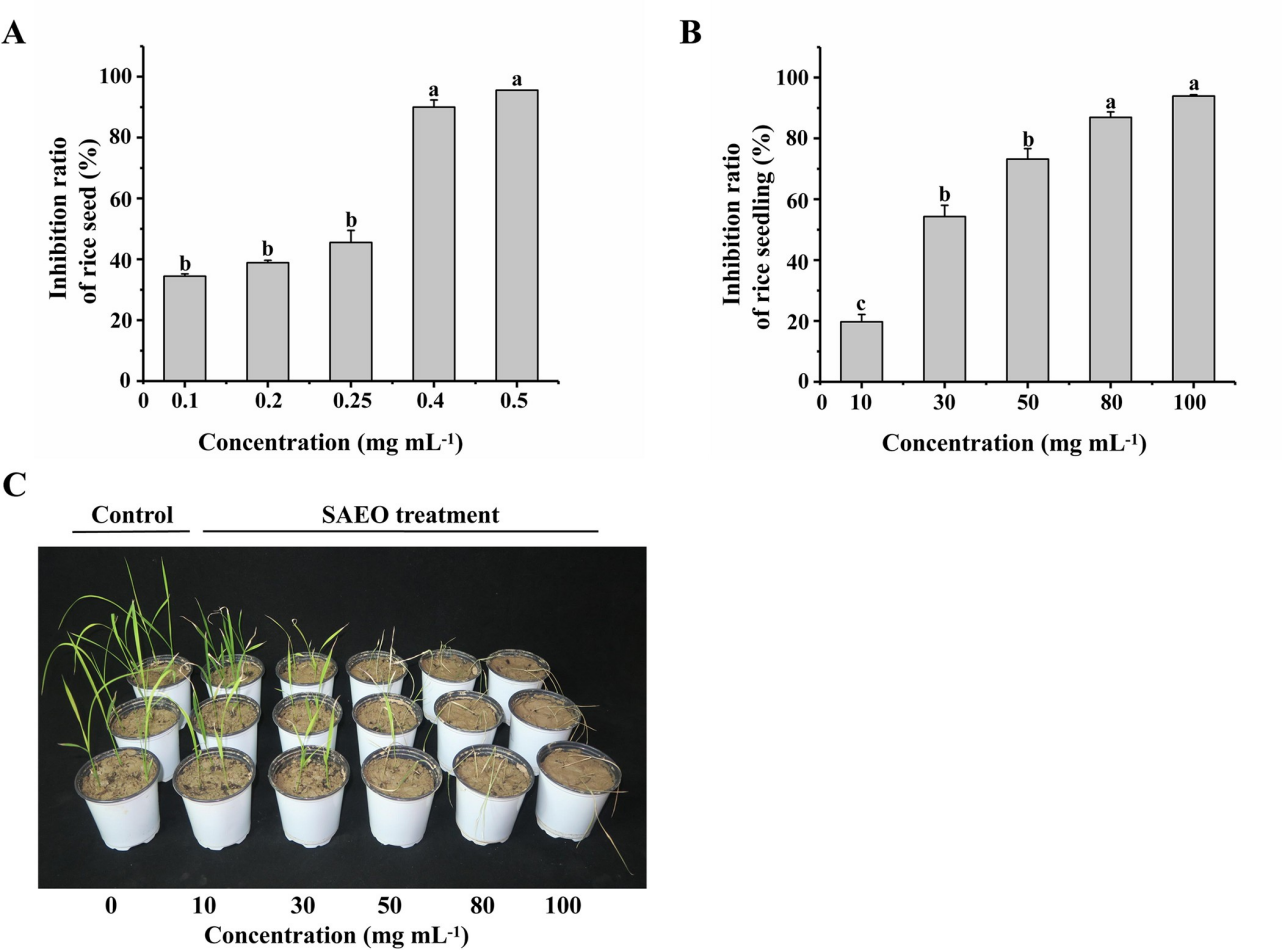
Safety evaluation of SAEO on rice. (**A**) Inhibitory effect of SAEO (0.1, 0.2, 0.25, 0.4, 0.5 mg mL^-1^) on rice seeds. (**B**) Inhibitory effect of SAEO (10, 30, 50, 80, 100 mg mL^-1^) on rice seedlings. (**C**) Inhibitory effect of different concentrations of SAEO (0, 10, 30, 50, 80, 100 mg mL^-1^) on rice.

To verify whether SAEO was safe for crops, we evaluated the safety of rice under SAEO stress (Fig 4). The inhibitory ratios of SAEO at concentrations of 0.1 and 0.5 mg mL^-1^ on rice at the germination stage were 34.44% and 95.56% respectively (Fig 4A). When the same concentration, the result showed nearly herbicidal effects on the barnyard grass seed. Hence, SAEO is relatively non-safe for rice seed. At the 2—leaf stage, the inhibition ratio was 19.71% when the concentration of SAEO was 10 mg mL^-1^, and 93.92% when the concentration was 100 mg mL^-1^ (Fig 4B). However, when SAEO treatment were performed by 10 mg mL^-1^, could inhibited barnyard grass seedling by reach approximately 80%. Therefore, SAEO is relatively safe for rice seedlings at around 10 mg mL^-1^. The inhibitory effect of SAEO on rice are shown in Fig 4C. The EC_50_ of SAEO to rice seed germination and seedling stage were 0.1831 mg mL^-1^ and 24.9431 mg mL^-1^, respectively (S4 Table). Results showed that the tolerance of rice to SAEO at seedling stage, was higher than that of *E*. *crus-galli* at the same concentration.

### SAEO inhibits the activities of defense enzymes in *E*. *crus-galli* and rice

**Fig 5:**
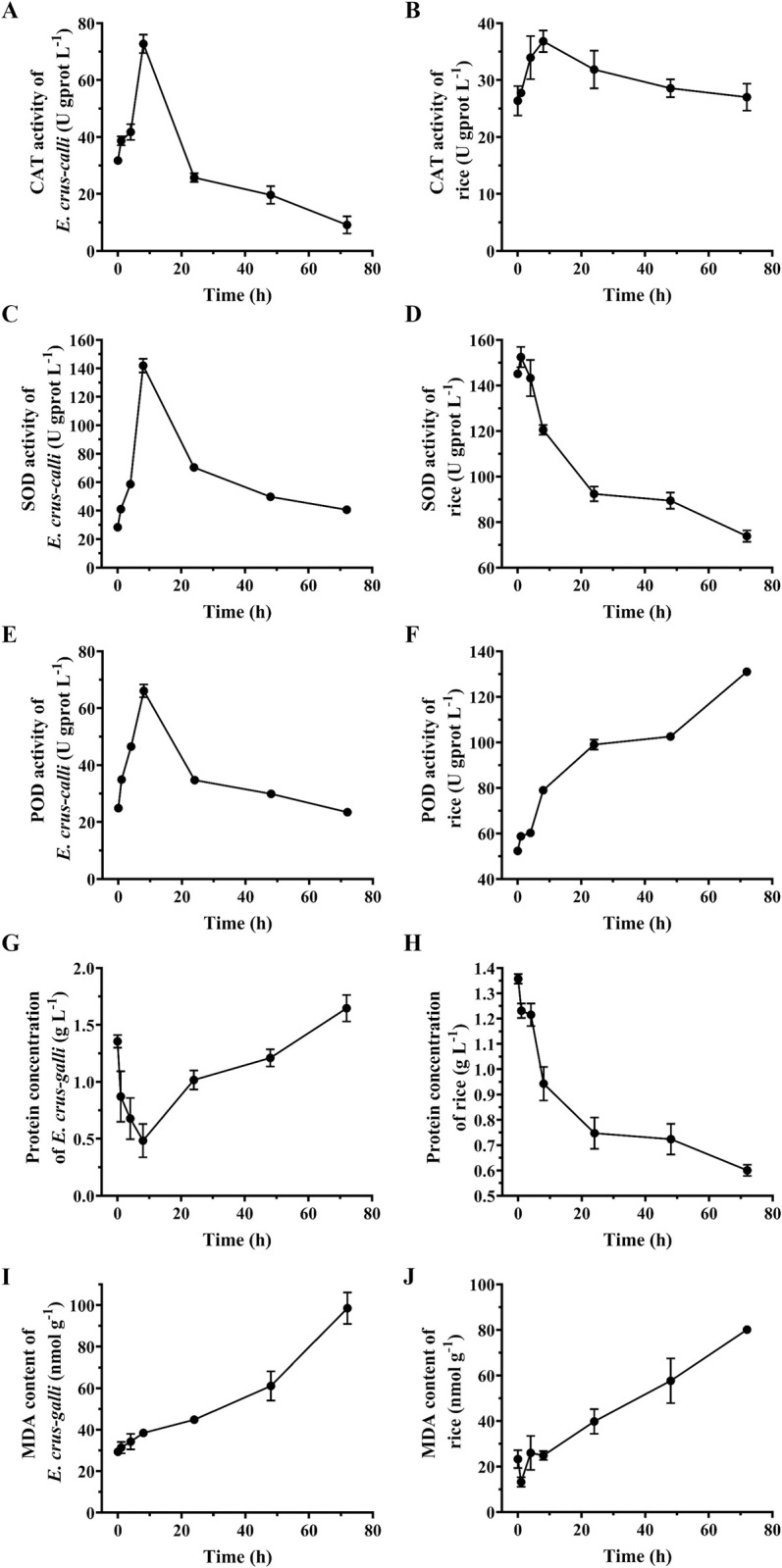
Effect of SAEO on the activities of defense enzymes and content of MDA in *E*. *crus-galli* and rice. Effects of SAEO on (**A**) CAT activity in *E*. *crus-galli* (**B**) CAT activity in rice (**C**) SOD activity in *E*. *crus-galli* (**D**) SOD activity in rice, (**E**) POD activity in *E*. *crus-galli* (**F**) POD activity in rice (**G**) TP activity in *E*. *crus-galli* (**H**) TP activity in rice, (**I**) MDA content in *E*. *crus-gall* (**J**) MDA content in rice.

The CAT activity in *E*. *crus-galli* and rice treated with SAEO first showed an increasing trend, then a decreasing trend (Fig 5). CAT activity in *E*. *crus-galli* was 31.753 U gprot^-1^ at 0 h, reached a peak value of 72.744 U gprot^-1^ at 8 h and then decreased to 9.143 U gprot^-1^ at 72 h, which was lower than that of the control group (Fig 5A). The CAT activity in *E*. *crus-galli* increased by 56.35% (0–8 h, compared to control), and decreased (0–72 h, compared to the control) by 71.21%. CAT activity in rice was 26.368 U gprot^-1^ at 0 h, reached a peak value of 36.828 U gprot^-1^ at 8h and then decreased to 27.009 U gprot^-1^ at 72 h, which was higher than that of the control group (Fig 5B). The CAT activity in rice increased by 28.4% (0–8 h, compared to control), and decreased (0–72 h, compared to the control) by -2.43% (Fig 5B).

*E*. *crus-galli* SOD activity was 41.103 U gprot^-1^ at 1 h, reached a peak value of 141.891 U gprot^-1^ at 8 h and then decreased to 39.199 U gprot^-1^ at 72 h (Fig 5C). SOD activity of rice was 145.13 U gprot^-1^ at 0 h, reached a peak value of 152.461 U gprot^-1^ at 1 h and then decreased to 73.831 U gprot^-1^ at 72 h (Fig 5D).

POD activity in *E*. *crus-galli* was 34.924 U gprot^-1^ at 1 h, reached a peak value of 66.075 U gprot^-1^ at 8 h and then decreased to 23.482 U gprot^-1^ at 72 h, which was nearly the same as that of the control group (Fig 5E). POD activity in rice was 52.32 U gprot^-1^ at 0 h, reached a peak value of 131.026 U gprot^-1^ at 72 h (Fig 5F).

The TP content in *E*. *crus-galli* was 1.356 gprot L^-1^ at 0 h, decreased to the lowest value of 0.484 gprot L^-1^ at 8 h, and gradually increased to 1.647 gprot L^-1^ between 8 h to 72 h (Fig 5G). The rice TP content was 1.357 gprot L^-1^ at 0 h, which decreased to the lowest value of 0.601 gprot L^-1^ at 72 h (Fig 5H). The relative activities of CAT, POD and SOD were calculated based on the TP.

As shown in Fig 5I and 5F, the content of MDA was significantly higher in *E*. *crus-galli* and rice exposed of SAEO. The MDA content in *E*. *crus-galli* was 29.33 nmol g^-1^ at 0 h, and gradually increased to 98.57 nmol g^-1^ between 0 h to 72 h. The MDA content in *E*. *crus-galli* increased by 336% (0–72 h, compared to control). The MDA content in rice was 23.24 nmol g^-1^ at 0 h, and gradually increased to 80.11 nmol g^-1^ between 0 h to 72 h. The MDA content in rice increased by 344.7% (0–72 h, compared to control).

## Discussion

In this study, we evaluated the phytotoxicities of 13 essential oils to find a possible application in the biocontrol of weeds. Among them, the SAEO were the best. Our findings showed a remarkable phytotoxic activity of SAEO against *E*. *crus-galli* in both Petri dish and pot experiments, in terms of reduction in germination and growth in a dose-dependent manner. Similar to our results, rice leaf essential oil was shown to have had phytotoxicity to *E*. *crus-galli* (IC_50_ = 964.3 ug mL^-1^), with the inhibitory effect proportional to the applied doses of oil [21]. Also, the essential oil from *Cymbopogon citratus* was reported to have significantly reduced the seed germination, root length and shoot lengths of *E*. *crus-galli* at 8 μL concentration [22]. The germination of *E*. *crus-galli* was reported to have been reduced by 92% in response to treatment with 0.5 mg mL^-1^ *Satureja montana* essential oil [23]. *Thymbra capitata* essential oil (2 μL mL^-1^) was also shown to have inhibited the germination of *E*. *crus-galli* seeds [24]. These show that SAEO has the potential to be developed as a herbicide against *E*. *crus-galli* in fields.

In our study, results showed that the main component of the essential oils extracted from *S*. *aromaticum* was eugenol. The recognized chemical composition of SAEO by GC-MS analysis was mostly consistent with findings of Ulanowska M and Olas B [25] and Hu Q [26]. Accordingly, the observed growth phytotoxic effect of SAEO could be attributed to the presence of the monoterpenes. Several studies have demonstrated that the EOs and their constituents, especially monoterpenes, are phytotoxic in nature. [27–30] Results from the plant bioassay, showed that eugenol, α-caryophyllene and β-caryophyllene had inhibitory effects on *E*. *crus-gall*i, but eugenol had the strongest inhibitory effect than the others. However, the phytotoxicity of individual monoterpenes was not higher than that of SAEO-165 and indicated that various monoterpenes may have a combined synergistic effect.

The safety of herbicides should not be ignored in the development process. In our research, SAEO exhibited greater toxicity against the weed *E*. *crus-galli* than the crop rice (Fig 4D), hence, the oil makes it worth exploiting for the management of weeds in the agricultural fields. This characteristic of the oil could reduce the chemical burden on agricultural systems since the EOs are biodegradable and EO compounds have a high structural diversity [31, 32]. However, before recommending this oil as a viable option for the sustainable management of weeds in agricultural systems, some non-negligible factors need to be ascertained, such as their low solubility, volatility, and their impact on nontarget organisms [33–35]. Nevertheless, the solubility, stability and efficacy of EOs can be enhanced by encapsulation [36, 37].

The antioxidant enzyme system in plants influence their physiological functions such as growth, development, stress defense and response and metabolic processes [38]. The effect of essential oils on the antioxidant enzyme system in plants have been evaluated in past studies [39, 40]. We therefore evaluated the effect of SAEO on the activities of antioxidant enzymes (CAT, SOD and POD) and oxidative stress markers in *E*. *crus-galli* and rice. The results indicated excessive generation of ROS and oxidative damage in the plant. In fact, ROS generation resulting in oxidative damage has been suggested as one of the mechanism of action of allelochemicals [41]. In the ROS system, SODs act as the first line of defense against ROS, dismutating superoxide to H_2_O_2_ and CAT subsequently detoxify H_2_O_2_ [42]. In our study, the imbalance between production and scavenging of ROS after stress in the two plants, resulting in different levels of oxidative stress in the cells, may be the key to the relative safety of the crops. Further, under stress conditions, primarily, plasma membrane reflected by accumulation of MDA after lipid peroxidation [43]. SAEO induced excessive generation of ROS suggests a similar mechanism of action leading to oxidative stress and ultimately tissue damage in *E*. *crus-galli*. Our results indicate that the application of SAEO may lead to a rapid accumulation of ROS in barnyard grass than in rice, indicating that SAEO may be relatively safe for rice. SAEO effectively inhibited the growth and development of barnyard grass seedlings, which might be related to its effect on the CAT activity in barnyard grass. This however needs further research.

In conclusion, this study showed that among 13 plants essential oils studied, SAEO had a high inhibitory activity against *E*. *crus-galli* in a dose-dependent manner. The SAEO was collected and purified by vacuum distillation under different conditions, and that which was collected at 165°C (SAEO—165) had the strongest inhibitory activity. GC-MS and HPLC analyses showed that SAEO—165 contained three main compounds (eugenol, β-caryophyllene and α-caryophyllene) and also had an effect on the activities of antioxidant enzymes in *E*. *crus-galli*. Further, SAEO was relatively safe to rice plants at the 2—leaves—stage and may serve as an effective and safe post-emergence herbicide against *E*. *crus-galli* in rice production (Fig 5). Further work should focus on stable and safety formulations of SAEO and field experiments to evaluate its efficacy.

## Supporting information

S1 Fig Inhibitory effect of different concentrations (0, 1, 5, 10, 20, 30, 50, 80, 100 mg mL-1) of 13 essential oils on Echinochloa crus-galli. (*M*. *piperita* essential oil (MPEO) # 1, *S*. *aromaticum* essential oil (SAEO) # 2, *E*. *caryophyllata* essential oil (ECEO) # 3, *C*. *cassia* essential oil (CCEO) # 4, *Z*. essential oil (ZEO) # 5, *C*. *citratus* essential oil (CIEO) # 6, *C*. *camphora* essential oil (CAEO) # 7, *C*. *annuumlinn* essential oil (CNEO) # 8, *C*. *sinensis* essential oil (CSEO) # 9, *A*. *caruifolia* essential oil (ACEO) # 10, *C*. *sinensis* essential oil (CEEO) # 11, *C*. *limon* essential oil # (CLEO) 12, and *C*. *reticulata* essential oil (CREO) # 13).
(TIF)

S2 Fig Inhibitory effect of 2 fraction distillations SAEO on Echinochloa crus-galli. A: Inhibitory effect of 10 mg mL^-1^ fractions distillation (165, 170 and 180°C) SAEO on *Echinochloa crus-galli*. B: Inhibitory effect of 30 mg mL^-1^ fractions distillation (165, 170 and 180°C) SAEO on *Echinochloa crus-galli*. C: Inhibitory effect of 50 mg mL^-1^ fractions distillation (165, 170 and 180°C) SAEO on *Echinochloa crus-galli*. D: Inhibitory effect of 10 mg mL^-1^ fractions distillation (164, 169 and 175°C) SAEO on *Echinochloa crus-galli*. E: Inhibitory effect of 30 mg mL^-1^ fractions distillation (164, 169 and 175°C) SAEO on *Echinochloa crus-galli*. F: Inhibitory effect of 50 mg mL^-1^ fractions distillation (164, 169 and 175°C) SAEO on *Echinochloa crus-galli*.
(TIF)

S1 Table The EC50 of SAEO at the E. crus-galli. The data of inhibition rate of *E*. *crus-galli* treated with SAEO(seed: control, 0.1, 0.2, 0.25, 0.4, 0.5 mg mL^-1^, seedling: control, 1, 5, 10, 20, 30 mg mL^-1^).
(DOCX)

S2 Table The EC50 of compounds at the E. crus-galli. The data of inhibition rate of *E*. *crus-galli* treated with eugenol, α-caryophyllene and β-caryophyllene(seedling: control, 1, 5, 10, 20, 30 mg mL^-1^).
(DOCX)

S3 Table The eugenol contents of SAEO from HPLC analysis. The contents of eugenol purified from different SAEO fraction.
(DOCX)

S4 Table The EC50 of SAEO at the rice. The data of inhibition rate of rice treated with three compounds (seedling: control, 10, 30, 50, 80, 100 mg mL^-1^).
(DOCX)

S5 Table ANOVA tables and comparing means graphs. ANOVA table and SNK multiple comparison result of inhibitory effect of SAEO and 13 plant oils on *E*. *crusgalli* seedling.
(DOCX)

S1 File The data presented in this study. (DOCX)
