# Supplementary figures and images for: Inhibitory activities of essential oils from Syzygium aromaticum inhibition of Echinochloa crus-galli
Source: PLoS One. 2024 Jun 21;19(6):e0304863. doi: 10.1371/journal.pone.0304863 (PMC11192376; doi:10.1371/journal.pone.0304863)

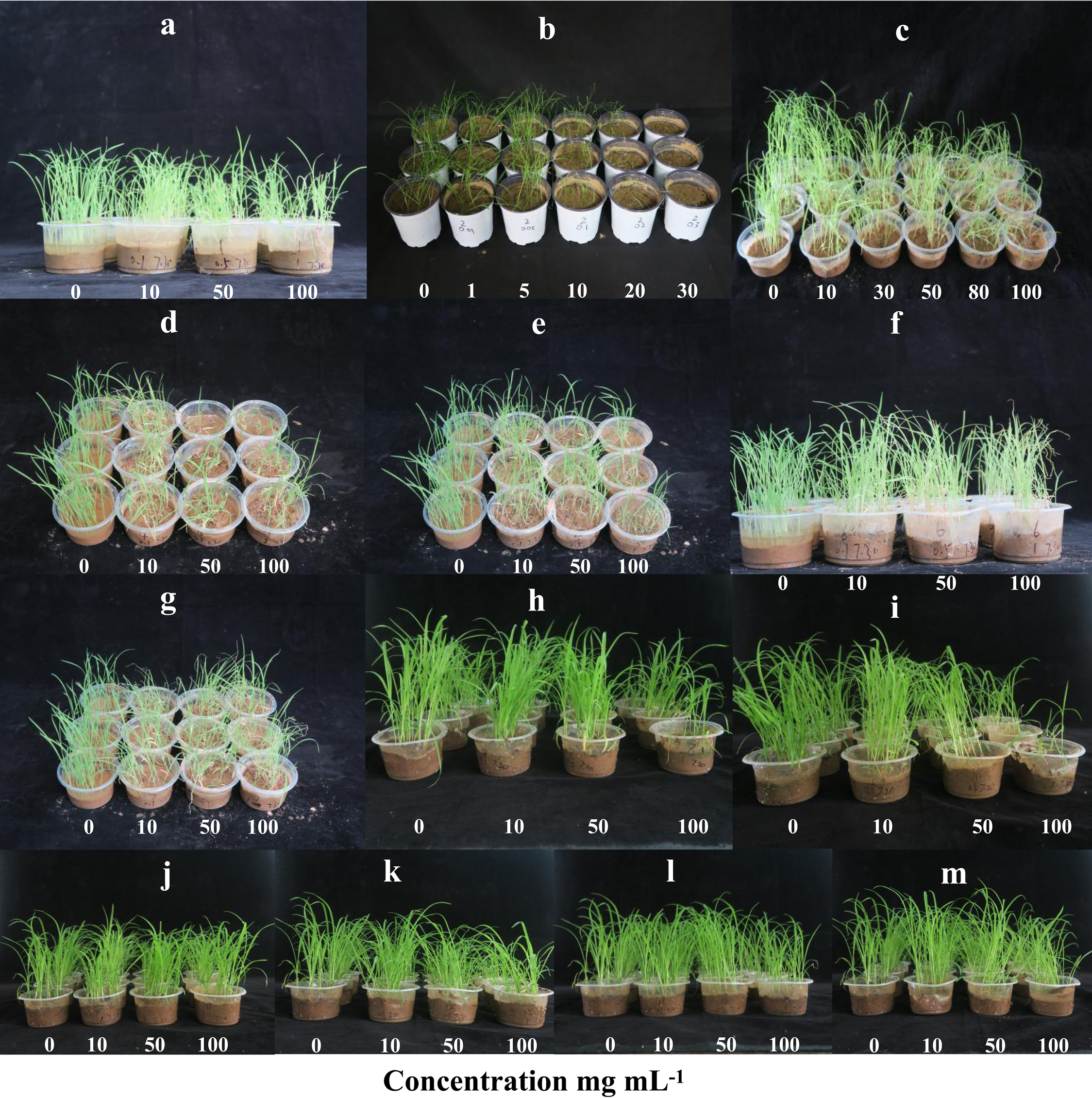

Supplement: S1 Fig — (M. piperita essential oil (MPEO) # 1, S. aromaticum essential oil (SAEO) # 2, E. caryophyllata essential oil (ECEO) # 3, C. cassia essential oil (CCEO) # 4, Z. essential oil (ZEO) # 5, C. citratus essential oil (CIEO) # 6, C. camphora essential oil (CAEO) # 7, C. annuumlinn essential oil (CNEO) # 8, C. sinensis essential oil (CSEO) # 9, A. caruifolia essential oil (ACEO) # 10, C. sinensis essential oil (CEEO) # 11, C. limon essential oil # (CLEO) 12, and C. reticulata essential oil (CREO) # 13). (TIF) [file pone.0304863.s001.tif]

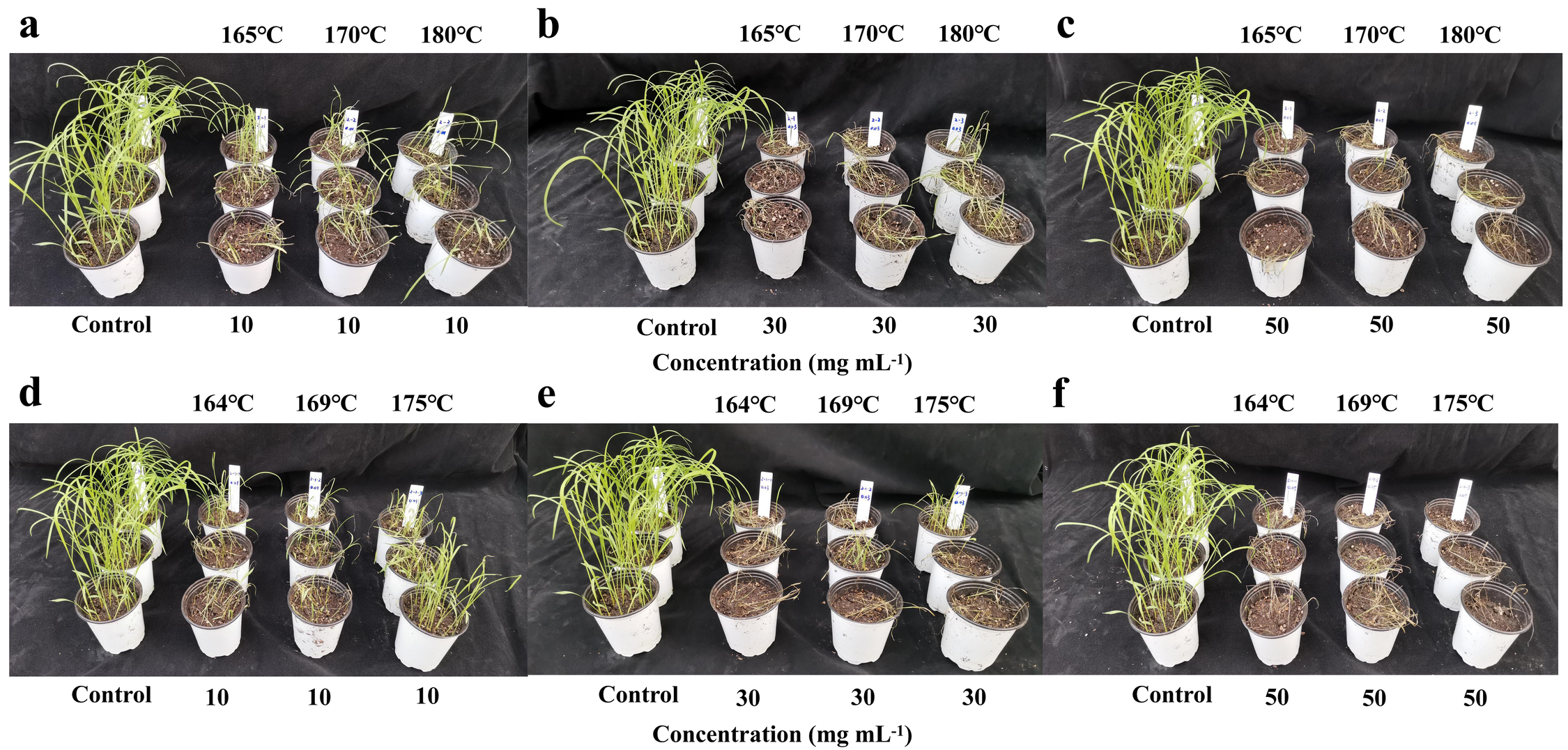

Supplement: S2 Fig — A: Inhibitory effect of 10 mg mL-1 fractions distillation (165, 170 and 180°C) SAEO on Echinochloa crus-galli. B: Inhibitory effect of 30 mg mL-1 fractions distillation (165, 170 and 180°C) SAEO on Echinochloa crus-galli. C: Inhibitory effect of 50 mg mL-1 fractions distillation (165, 170 and 180°C) SAEO on Echinochloa crus-galli. D: Inhibitory effect of 10 mg mL-1 fractions distillation (164, 169 and 175°C) SAEO on Echinochloa crus-galli. E: Inhibitory effect of 30 mg mL-1 fractions distillation (164, 169 and 175°C) SAEO on Echinochloa crus-galli. F: Inhibitory effect of 50 mg mL-1 fractions distillation (164, 169 and 175°C) SAEO on Echinochloa crus-galli. (TIF) [file pone.0304863.s002.tif]
