# Supplementary material for: Inhibitory activities of essential oils from Syzygium aromaticum inhibition of Echinochloa crus-galli
Source: PLoS One. 2024 Jun 21;19(6):e0304863. doi: 10.1371/journal.pone.0304863 (PMC11192376; doi:10.1371/journal.pone.0304863)
Supplement: S1 Table — The data of inhibition rate of E. crus-galli treated with SAEO(seed: control, 0.1, 0.2, 0.25, 0.4, 0.5 mg mL-1, seedling: control, 1, 5, 10, 20, 30 mg mL-1). (DOCX) [file pone.0304863.s003.docx]

| **Table S1 The EC_50_ of SAEO at the *E. crus-galli*** | | | | | |
| --- | --- | --- | --- | --- | --- |
| **Stage** | **Regression**  **formula** | **Related**  **coefficient** | **EC_50_**  **(mg mL^-1^)** | **95% confidence**  **limits** | **P-value** |
| Germination | Y=8.6871+4.5526X | 0.9285 | 0.1549 | 0.1107~0.2168 | 0.0227 |
| Seedling | Y=3.9715+1.7495X | 0.9971 | 3.8717 | 3.4383~4.3641 | 0.0002 |
