# Supplementary material for: Inhibitory activities of essential oils from Syzygium aromaticum inhibition of Echinochloa crus-galli
Source: PLoS One. 2024 Jun 21;19(6):e0304863. doi: 10.1371/journal.pone.0304863 (PMC11192376; doi:10.1371/journal.pone.0304863)
Supplement: S2 Table — The data of inhibition rate of E. crus-galli treated with eugenol, α-caryophyllene and β-caryophyllene(seedling: control, 1, 5, 10, 20, 30 mg mL-1). (DOCX) [file pone.0304863.s004.docx]

| **Table S2 The EC_50_ of compounds at the *E. crus-galli*** | | | | | |
| --- | --- | --- | --- | --- | --- |
| **Essential compound** | **Regression**  **formula** | **Related**  **coefficient** | **EC_50_**  **(mg mL^-1^)** | **95% confidence limits** | **P-value** |
| Eugenol | Y=3.9309+1.7548X | 0.9493 | 4.0666 | 2.4355~6.7899 | 0.0136 |
| α-Caryophyllene | Y=4.0869+0.7370X | 0.8657 | 17.3361 | 6.7764~44.3513 | 0.0579 |
| β-Caryophyllene | Y=3.5134+0.7488X | 0.9461 | 96.6599 | 32.9046~283.9456 | 0.0149 |
