# Supplementary material for: Inhibitory activities of essential oils from Syzygium aromaticum inhibition of Echinochloa crus-galli
Source: PLoS One. 2024 Jun 21;19(6):e0304863. doi: 10.1371/journal.pone.0304863 (PMC11192376; doi:10.1371/journal.pone.0304863)
Supplement: S3 Table — The contents of eugenol purified from different SAEO fraction. (DOCX) [file pone.0304863.s005.docx]

| **Table S3** **The eugenol contents of SAEO from HPLC analysis** | | | | | |
| --- | --- | --- | --- | --- | --- |
| **Sample Name**  **(LIA)** | **Retention Time**  **(min)** | **Concentration**  **(mg/L)** | | **Peak Area**  **（A**） | **Peak Area**  **Percentage**（A%） |
| 164℃ | 5.5 | 1915581 | 50488 | | 50.488 |
| 165℃ | 5.512 | 2798930 | 73770 | | 73.77 |
| 169℃ | 5.497 | 2614384 | 68906 | | 68.906 |
| 170℃ | 5.529 | 3162939 | 83364 | | 83.364 |
| 175℃ | 5.494 | 2572686 | 67807 | | 67.807 |
| 180℃ | 5.533 | 3122797 | 82306 | | 82.306 |
