# Supplementary material for: Inhibitory activities of essential oils from Syzygium aromaticum inhibition of Echinochloa crus-galli
Source: PLoS One. 2024 Jun 21;19(6):e0304863. doi: 10.1371/journal.pone.0304863 (PMC11192376; doi:10.1371/journal.pone.0304863)
Supplement: S4 Table — The data of inhibition rate of rice treated with three compounds (seedling: control, 10, 30, 50, 80, 100 mg mL-1). (DOCX) [file pone.0304863.s006.docx]

| **Table S4 The EC50 of SAEO at the rice** | | | | | |
| --- | --- | --- | --- | --- | --- |
| **Stage** | **Regression**  **formula** | **Related**  **coefficient** | **EC_50_**  **(mg mL^-1^)** | **95% confidence**  **limits** | **P-value** |
| Germination | Y=7.3494+3.1862X | 0.8916 | 0.1831 | 0.1262~0.2655 | 0.0421 |
| Seedling | Y=1.7506+2.3261X | 0.9939 | 24.9431 | 22.104~28.1468 | 0.0006 |
