# Supplementary material for: Inhibitory activities of essential oils from Syzygium aromaticum inhibition of Echinochloa crus-galli
Source: PLoS One. 2024 Jun 21;19(6):e0304863. doi: 10.1371/journal.pone.0304863 (PMC11192376; doi:10.1371/journal.pone.0304863)
Supplement: S5 Table — ANOVA table and SNK multiple comparison result of inhibitory effect of SAEO and 13 plant oils on E. crusgalli seedling. (DOCX) [file pone.0304863.s007.docx]

**Table S5-1.** ANOVA table of inhibitory effect of SAEO on *E. crusgalli* seedling.

| ANOVA table of inhibitory effect of SAEO on *E. crusgalli* seedling | | | | | |
| --- | --- | --- | --- | --- | --- |
| Source | Sum of Squares | Degree of freedom | Mean square | F-value | P-value |
| Between treatments | 8234.6231 | 4 | 2058.6558 | 45.724 | 0.0004 |
| In treatments | 225.1177 | 5 | 45.0235 |  |  |
| Total variation | 8459.7408 | 9 |  |  |  |

| SNK multiple comparison | | | | | | |
| --- | --- | --- | --- | --- | --- | --- |
| Concentration (mg mL^-1^) | average | 30 | 20 | 10 | 5 | 1 |
| 30 | 94.5604 |  | 0.331 | 0.0777 | 0.0028 | 0.0001 |
| 20 | 87.3392 | 7.2212 |  | 0.2824 | 0.0064 | 0.0002 |
| 10 | 79.2586 | 15.3018 | 8.0806 |  | 0.0182 | 0.0003 |
| 5 | 56.0944 | 38.4661 | 31.2449 | 23.1643 |  | 0.0017 |
| 1 | 15.2693 | 79.2912 | 72.07 | 63.9894 | 40.8251 |  |

*lower triangle is mean difference, upper triangle is significance level

| SNK multiple comparison result | | | | |
| --- | --- | --- | --- | --- |
| Concentration (mg mL^-1^) | average | 5%significant leve |  | 1%Extreme significance level |
| 30 | 94.5604 | a |  | A |
| 20 | 87.3392 | a |  | A |
| 10 | 79.2586 | a |  | AB |
| 5 | 56.0944 | b |  | B |
| 1 | 15.2693 | c |  | C |

**Table S5-2.** ANOVA table of inhibitory effect of SAEO on rice seedling.

| ANOVA table of inhibitory effect of SAEO on rice seedling | | | | | |
| --- | --- | --- | --- | --- | --- |
| Source | Sum of Squares | Degree of freedom | Mean square | F-value | P-value |
| Between treatments | 7094.7389 | 4 | 1773.6847 | 254.548 | 0.0001 |
| In treatments | 34.8399 | 5 | 6.968 |  |  |
| Total variation | 7129.5788 | 9 |  |  |  |

| SNK multiple comparison | | | | | | |
| --- | --- | --- | --- | --- | --- | --- |
| Concentration (mg mL^-1^) | average | 100 | 80 | 50 | 30 | 10 |
| 100 | 93.9169 |  | 0.0454 | 0.0006 | 0 | 0 |
| 80 | 86.9223 | 6.9946 |  | 0.0035 | 0.0001 | 0 |
| 50 | 73.1989 | 20.718 | 13.7234 |  | 0.0008 | 0 |
| 30 | 54.3098 | 39.6071 | 32.6125 | 18.8891 |  | 0 |
| 10 | 19.7124 | 74.2045 | 67.2099 | 53.4865 | 34.5974 |  |

*lower triangle is mean difference, upper triangle is significance level

| SNK multiple comparison result | | | | |
| --- | --- | --- | --- | --- |
| EOs | average | 5%significant leve |  | 1%Extreme significance level |
| 100 | 93.9169 | a |  | A |
| 80 | 86.9223 | b |  | A |
| 50 | 73.1989 | c |  | B |
| 30 | 54.3098 | d |  | C |
| 10 | 19.7124 | e |  | D |

**Table S5-3.** ANOVA table of inhibitory effect of 13 plant oils on *E. crusgalli* seedling.

| ANOVA table of inhibitory effect of 13 plant oils on *E. crusgalli* seedling | | | | | |
| --- | --- | --- | --- | --- | --- |
| Source | Sum of Squares | Degree of freedom | Mean square | F-value | P-value |
| Between treatments | 13000.1931 | 12 | 1083.3494 | 328.474 | 0.0001 |
| In treatments | 42.8757 | 13 | 3.2981 |  |  |
| Total variation | 13043.0688 | 25 |  |  |  |

| SNK multiple comparison | | | | | | |
| --- | --- | --- | --- | --- | --- | --- |
| Concentration (mg mL^-1^) | average | 30 | 20 | 10 | 5 | 1 |
| 30 | 95.6856 |  | 0 | 0 | 0 | 0 |
| 20 | 80.3494 | 15.3362 |  | 0 | 0 | 0 |
| 10 | 69.6593 | 26.0263 | 10.6901 |  | 0 | 0 |
| 5 | 35.876 | 59.8096 | 44.4734 | 33.7833 |  | 0 |
| 1 | 19.3292 | 76.3564 | 61.0202 | 50.3302 | 16.5468 |  |

| SNK multiple comparison | | | | | | | | | | | | | |
| --- | --- | --- | --- | --- | --- | --- | --- | --- | --- | --- | --- | --- | --- |
| EOs | average | SAEO | CCEO | ZEO | CAEO | ECEO | CIEO | MPEO | CNEO | AC | CSEO | CLEO | CREO |
| SAEO | 88.0783 |  | 0 | 0 | 0 | 0 | 0 | 0 | 0 | 0 | 0 | 0 | 0 |
| CCEO | 43.4164 | 44.6619 |  | 0 | 0 | 0 | 0 | 0 | 0 | 0 | 0 | 0 | 0 |
| ZEO | 27.3913 | 60.687 | 16.0251 |  | 0.0831 | 0.0643 | 0 | 0 | 0 | 0 | 0 | 0 | 0 |
| CAEO | 23.9823 | 64.096 | 19.4341 | 3.409 |  | 0.8131 | 0 | 0 | 0 | 0 | 0 | 0 | 0 |
| ECEO | 23.5441 | 64.5342 | 19.8723 | 3.8472 | 0.4382 |  | 0 | 0 | 0 | 0 | 0 | 0 | 0 |
| CIEO | 10.5769 | 77.5014 | 32.8394 | 16.8144 | 13.4054 | 12.9672 |  | 0.7056 | 0.3797 | 0.3685 | 0.1195 | 0.0809 | 0.0312 |
| MPEO | 9.8754 | 78.2028 | 33.5409 | 17.5159 | 14.1069 | 13.6686 | 0.7015 |  | 0.5776 | 0.5658 | 0.2046 | 0.1425 | 0.0571 |
| CNEO | 8.838 | 79.2403 | 34.5784 | 18.5533 | 15.1443 | 14.7061 | 1.739 | 1.0375 |  | 0.9612 | 0.4229 | 0.3098 | 0.136 |
| AC | 8.748 | 79.3303 | 34.6684 | 18.6433 | 15.2343 | 14.7961 | 1.8289 | 1.1275 | 0.09 |  | 0.426 | 0.318 | 0.1404 |
| CSEO | 7.2555 | 80.8228 | 36.1608 | 20.1358 | 16.7268 | 16.2886 | 3.3214 | 2.6199 | 1.5824 | 1.4925 |  | 0.7906 | 0.4221 |
| CLEO | 6.7633 | 81.315 | 36.6531 | 20.628 | 17.219 | 16.7808 | 3.8136 | 3.1122 | 2.0747 | 1.9847 | 0.4922 |  | 0.5576 |
| CREO | 5.6701 | 82.4082 | 37.7463 | 21.7212 | 18.3122 | 17.874 | 4.9068 | 4.2053 | 3.1679 | 3.0779 | 1.5854 | 1.0932 |  |
| CEEO | 4.0698 | 84.0085 | 39.3466 | 23.3215 | 19.9125 | 19.4743 | 6.5072 | 5.8057 | 4.7682 | 4.6782 | 3.1858 | 2.6935 | 1.6003 |

*lower triangle is mean difference, upper triangle is significance level

| SNK multiple comparison result | | | | |
| --- | --- | --- | --- | --- |
| EOs | average | 5%significant leve |  | 1%Extreme significance level |
| SAEO | 88.0783 | a |  | A |
| CCEO | 43.4164 | b |  | B |
| ZEO | 27.3913 | c |  | C |
| CAEO | 23.9823 | c |  | C |
| ECEO | 23.5441 | c |  | C |
| CIEO | 10.5769 | d |  | D |
| MPEO | 9.8754 | de |  | DE |
| CNEO | 8.838 | de |  | DE |
| AC | 8.748 | de |  | DE |
| CSEO | 7.2555 | def |  | DE |
| CLEO | 6.7633 | def |  | DE |
| CREO | 5.6701 | ef |  | DE |
