# Supplementary material for: Inhibitory activities of essential oils from Syzygium aromaticum inhibition of Echinochloa crus-galli
Source: PLoS One. 2024 Jun 21;19(6):e0304863. doi: 10.1371/journal.pone.0304863 (PMC11192376; doi:10.1371/journal.pone.0304863)
Supplement: S1 File — (DOCX) [file pone.0304863.s008.docx]

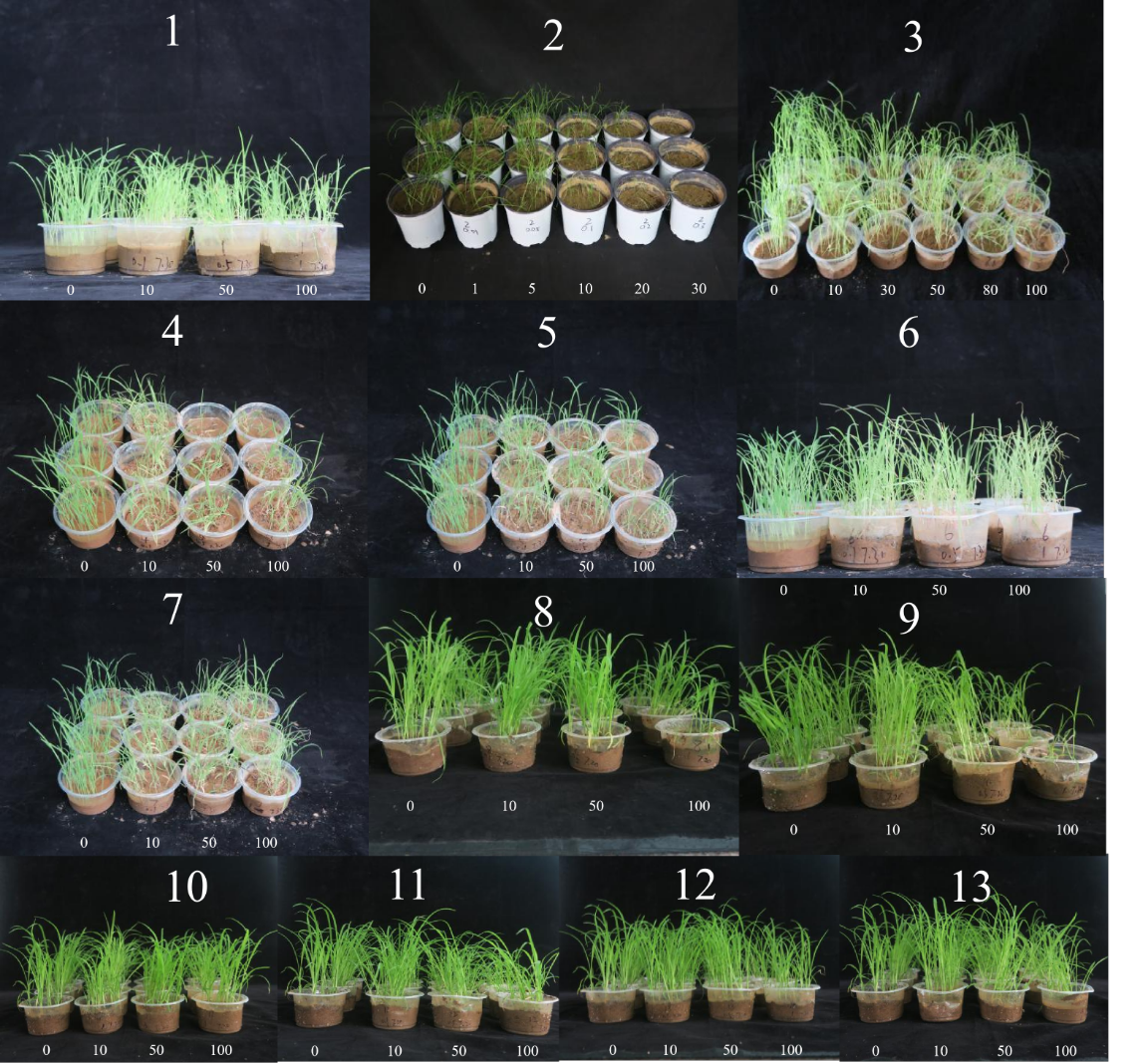


**Figture S1. Inhibitory effect of different concentrations** **(0, 1, 5, 10, 20, 30, 50, 80, 100 mg mL^-1^)** **of 13 essential oils on *Echinochloa crus-galli*.** (*M. piperita* essential oil (MPEO) # 1, *S. aromaticum* essential oil (SAEO) # 2, *E. caryophyllata* essential oil (ECEO) # 3, *C. cassia* essential oil (CCEO) # 4, *Z.* essential oil (ZEO) # 5, *C. citratus* essential oil (CIEO) # 6, *C. camphora* essential oil (CAEO) # 7, *C. annuumlinn* essential oil (CNEO) # 8, *C. sinensis* essential oil (CSEO) # 9, *A. caruifolia* essential oil (ACEO) # 10, *C. sinensis* essential oil (CEEO) # 11, *C. limon* essential oil # (CLEO) 12, and *C. reticulata* essential oil (CREO) # 13).


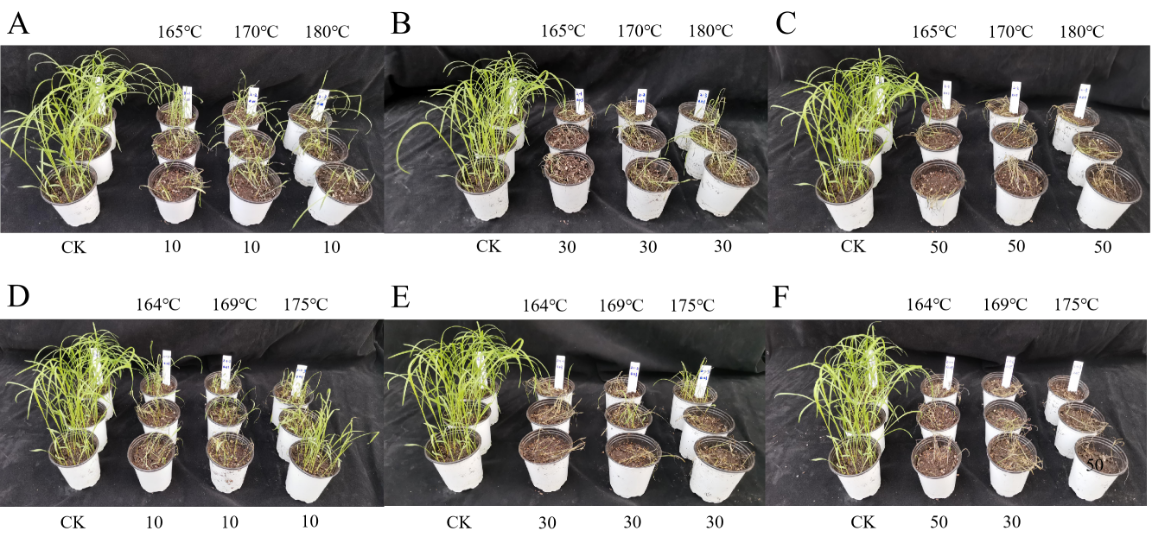


**Figture S2.** **Inhibitory effect of 2 fractions distillation LIA on** ***Echinochloa crus-galli*.** A: Inhibitory effect of 10 mg mL^-1^ fractions distillation (165, 170 and 180℃) SAEO on *Echinochloa crus-galli*. B: Inhibitory effect of 30 mg mL^-1^ fractions distillation (165, 170 and 180℃) SAEO on *Echinochloa crus-galli*. C: Inhibitory effect of 50 mg mL^-1^ fractions distillation (165, 170 and 180℃) SAEO on *Echinochloa crus-galli*. D: Inhibitory effect of 10 mg mL^-1^ fractions distillation (164, 169 and 175℃) SAEO on *Echinochloa crus-galli*. E: Inhibitory effect of 30 mg mL^-1^ fractions distillation (164, 169 and 175℃) SAEO on *Echinochloa crus-galli*. F: Inhibitory effect of 50 mg mL^-1^ fractions distillation (164, 169 and 175℃) SAEO on *Echinochloa crus-galli*. Of late, a number of synthetic chemicals have been tested and applied to counteract the negative impacts of weeds on crops

| **Table S1 The EC_50_ of SAEO at the *E. crus-galli*** | | | | | |
| --- | --- | --- | --- | --- | --- |
| **Stage** | **Regression**  **formula** | **Related**  **coefficient** | **EC_50_**  **(mg mL^-1^)** | **95% confidence**  **limits** | **P-value** |
| Germination | Y=8.6871+4.5526X | 0.9285 | 0.1549 | 0.1107~0.2168 | 0.0227 |
| Seedling | Y=3.9715+1.7495X | 0.9971 | 3.8717 | 3.4383~4.3641 | 0.0002 |

| **Table S2 The EC_50_ of compounds at the *E. crus-galli*** | | | | | |
| --- | --- | --- | --- | --- | --- |
| **Essential compound** | **Regression**  **formula** | **Related**  **coefficient** | **EC_50_**  **(mg mL^-1^)** | **95% confidence limits** | **P-value** |
| Eugenol | Y=3.9309+1.7548X | 0.9493 | 4.0666 | 2.4355~6.7899 | 0.0136 |
| α-Caryophyllene | Y=4.0869+0.7370X | 0.8657 | 17.3361 | 6.7764~44.3513 | 0.0579 |
| β-Caryophyllene | Y=3.5134+0.7488X | 0.9461 | 96.6599 | 32.9046~283.9456 | 0.0149 |

| **Table S3** **The eugenol contents of SAEO from HPLC analysis** | | | | | |
| --- | --- | --- | --- | --- | --- |
| **Sample Name**  **(LIA)** | **Retention Time**  **(min)** | **Concentration**  **(mg/L)** | | **Peak Area**  **（A**） | **Peak Area**  **Percentage**（A%） |
| 164℃ | 5.5 | 1915581 | 50488 | | 50.488 |
| 165℃ | 5.512 | 2798930 | 73770 | | 73.77 |
| 169℃ | 5.497 | 2614384 | 68906 | | 68.906 |
| 170℃ | 5.529 | 3162939 | 83364 | | 83.364 |
| 175℃ | 5.494 | 2572686 | 67807 | | 67.807 |
| 180℃ | 5.533 | 3122797 | 82306 | | 82.306 |

| **Table S4 The EC50 of SAEO at the rice** | | | | | |
| --- | --- | --- | --- | --- | --- |
| **Stage** | **Regression**  **formula** | **Related**  **coefficient** | **EC_50_**  **(mg mL^-1^)** | **95% confidence**  **limits** | **P-value** |
| Germination | Y=7.3494+3.1862X | 0.8916 | 0.1831 | 0.1262~0.2655 | 0.0421 |
| Seedling | Y=1.7506+2.3261X | 0.9939 | 24.9431 | 22.104~28.1468 | 0.0006 |

**Table S5-1.** ANOVA table of inhibitory effect of SAEO on *E. crusgalli* seedling.

| ANOVA table of inhibitory effect of SAEO on *E. crusgalli* seedling | | | | | |
| --- | --- | --- | --- | --- | --- |
| Source | Sum of Squares | Degree of freedom | Mean square | F-value | P-value |
| Between treatments | 8234.6231 | 4 | 2058.6558 | 45.724 | 0.0004 |
| In treatments | 225.1177 | 5 | 45.0235 |  |  |
| Total variation | 8459.7408 | 9 |  |  |  |

| SNK multiple comparison | | | | | | |
| --- | --- | --- | --- | --- | --- | --- |
| Concentration (mg mL^-1^) | average | 30 | 20 | 10 | 5 | 1 |
| 30 | 94.5604 |  | 0.331 | 0.0777 | 0.0028 | 0.0001 |
| 20 | 87.3392 | 7.2212 |  | 0.2824 | 0.0064 | 0.0002 |
| 10 | 79.2586 | 15.3018 | 8.0806 |  | 0.0182 | 0.0003 |
| 5 | 56.0944 | 38.4661 | 31.2449 | 23.1643 |  | 0.0017 |
| 1 | 15.2693 | 79.2912 | 72.07 | 63.9894 | 40.8251 |  |

*lower triangle is mean difference, upper triangle is significance level

| SNK multiple comparison result | | | | |
| --- | --- | --- | --- | --- |
| Concentration (mg mL^-1^) | average | 5%significant leve |  | 1%Extreme significance level |
| 30 | 94.5604 | a |  | A |
| 20 | 87.3392 | a |  | A |
| 10 | 79.2586 | a |  | AB |
| 5 | 56.0944 | b |  | B |
| 1 | 15.2693 | c |  | C |

**Table S5-2.** ANOVA table of inhibitory effect of SAEO on rice seedling.

| ANOVA table of inhibitory effect of SAEO on rice seedling | | | | | |
| --- | --- | --- | --- | --- | --- |
| Source | Sum of Squares | Degree of freedom | Mean square | F-value | P-value |
| Between treatments | 7094.7389 | 4 | 1773.6847 | 254.548 | 0.0001 |
| In treatments | 34.8399 | 5 | 6.968 |  |  |
| Total variation | 7129.5788 | 9 |  |  |  |

| SNK multiple comparison | | | | | | |
| --- | --- | --- | --- | --- | --- | --- |
| Concentration (mg mL^-1^) | average | 100 | 80 | 50 | 30 | 10 |
| 100 | 93.9169 |  | 0.0454 | 0.0006 | 0 | 0 |
| 80 | 86.9223 | 6.9946 |  | 0.0035 | 0.0001 | 0 |
| 50 | 73.1989 | 20.718 | 13.7234 |  | 0.0008 | 0 |
| 30 | 54.3098 | 39.6071 | 32.6125 | 18.8891 |  | 0 |
| 10 | 19.7124 | 74.2045 | 67.2099 | 53.4865 | 34.5974 |  |

*lower triangle is mean difference, upper triangle is significance level

| SNK multiple comparison result | | | | |
| --- | --- | --- | --- | --- |
| EOs | average | 5%significant leve |  | 1%Extreme significance level |
| 100 | 93.9169 | a |  | A |
| 80 | 86.9223 | b |  | A |
| 50 | 73.1989 | c |  | B |
| 30 | 54.3098 | d |  | C |
| 10 | 19.7124 | e |  | D |

**Table S5-3.** ANOVA table of inhibitory effect of 13 plant oils on *E. crusgalli* seedling.

| ANOVA table of inhibitory effect of 13 plant oils on *E. crusgalli* seedling | | | | | |
| --- | --- | --- | --- | --- | --- |
| Source | Sum of Squares | Degree of freedom | Mean square | F-value | P-value |
| Between treatments | 13000.1931 | 12 | 1083.3494 | 328.474 | 0.0001 |
| In treatments | 42.8757 | 13 | 3.2981 |  |  |
| Total variation | 13043.0688 | 25 |  |  |  |

| SNK multiple comparison | | | | | | |
| --- | --- | --- | --- | --- | --- | --- |
| Concentration (mg mL^-1^) | average | 30 | 20 | 10 | 5 | 1 |
| 30 | 95.6856 |  | 0 | 0 | 0 | 0 |
| 20 | 80.3494 | 15.3362 |  | 0 | 0 | 0 |
| 10 | 69.6593 | 26.0263 | 10.6901 |  | 0 | 0 |
| 5 | 35.876 | 59.8096 | 44.4734 | 33.7833 |  | 0 |
| 1 | 19.3292 | 76.3564 | 61.0202 | 50.3302 | 16.5468 |  |

| SNK multiple comparison | | | | | | | | | | | | | |
| --- | --- | --- | --- | --- | --- | --- | --- | --- | --- | --- | --- | --- | --- |
| EOs | average | SAEO | CCEO | ZEO | CAEO | ECEO | CIEO | MPEO | CNEO | AC | CSEO | CLEO | CREO |
| SAEO | 88.0783 |  | 0 | 0 | 0 | 0 | 0 | 0 | 0 | 0 | 0 | 0 | 0 |
| CCEO | 43.4164 | 44.6619 |  | 0 | 0 | 0 | 0 | 0 | 0 | 0 | 0 | 0 | 0 |
| ZEO | 27.3913 | 60.687 | 16.0251 |  | 0.0831 | 0.0643 | 0 | 0 | 0 | 0 | 0 | 0 | 0 |
| CAEO | 23.9823 | 64.096 | 19.4341 | 3.409 |  | 0.8131 | 0 | 0 | 0 | 0 | 0 | 0 | 0 |
| ECEO | 23.5441 | 64.5342 | 19.8723 | 3.8472 | 0.4382 |  | 0 | 0 | 0 | 0 | 0 | 0 | 0 |
| CIEO | 10.5769 | 77.5014 | 32.8394 | 16.8144 | 13.4054 | 12.9672 |  | 0.7056 | 0.3797 | 0.3685 | 0.1195 | 0.0809 | 0.0312 |
| MPEO | 9.8754 | 78.2028 | 33.5409 | 17.5159 | 14.1069 | 13.6686 | 0.7015 |  | 0.5776 | 0.5658 | 0.2046 | 0.1425 | 0.0571 |
| CNEO | 8.838 | 79.2403 | 34.5784 | 18.5533 | 15.1443 | 14.7061 | 1.739 | 1.0375 |  | 0.9612 | 0.4229 | 0.3098 | 0.136 |
| AC | 8.748 | 79.3303 | 34.6684 | 18.6433 | 15.2343 | 14.7961 | 1.8289 | 1.1275 | 0.09 |  | 0.426 | 0.318 | 0.1404 |
| CSEO | 7.2555 | 80.8228 | 36.1608 | 20.1358 | 16.7268 | 16.2886 | 3.3214 | 2.6199 | 1.5824 | 1.4925 |  | 0.7906 | 0.4221 |
| CLEO | 6.7633 | 81.315 | 36.6531 | 20.628 | 17.219 | 16.7808 | 3.8136 | 3.1122 | 2.0747 | 1.9847 | 0.4922 |  | 0.5576 |
| CREO | 5.6701 | 82.4082 | 37.7463 | 21.7212 | 18.3122 | 17.874 | 4.9068 | 4.2053 | 3.1679 | 3.0779 | 1.5854 | 1.0932 |  |
| CEEO | 4.0698 | 84.0085 | 39.3466 | 23.3215 | 19.9125 | 19.4743 | 6.5072 | 5.8057 | 4.7682 | 4.6782 | 3.1858 | 2.6935 | 1.6003 |

*lower triangle is mean difference, upper triangle is significance level

| SNK multiple comparison result | | | | |
| --- | --- | --- | --- | --- |
| EOs | average | 5%significant leve |  | 1%Extreme significance level |
| SAEO | 88.0783 | a |  | A |
| CCEO | 43.4164 | b |  | B |
| ZEO | 27.3913 | c |  | C |
| CAEO | 23.9823 | c |  | C |
| ECEO | 23.5441 | c |  | C |
| CIEO | 10.5769 | d |  | D |
| MPEO | 9.8754 | de |  | DE |
| CNEO | 8.838 | de |  | DE |
| AC | 8.748 | de |  | DE |
| CSEO | 7.2555 | def |  | DE |
| CLEO | 6.7633 | def |  | DE |
| CREO | 5.6701 | ef |  | DE |
